# Supplementary material for: Identification of photoperiod-induced specific miRNAs in the adrenal glands of Sunite sheep (Ovis aries)
Source: Front Vet Sci. 2022 Jul 22;9:888207. doi: 10.3389/fvets.2022.888207 (PMC9354845; doi:10.3389/fvets.2022.888207)
Supplement: Supplementary file 1 [file Data_Sheet_1.ZIP › Supplementary file/miRDeep2 file.zip!/miRDeep2 file/Know_Structure/SP42A2/oar-mir-299.pdf]

miRBase precursor : oar-mir-299
Total read count : 248
oar-miR-299-5p read count : 239
oar-miR-299-3p read count : 8
remaining reads : 1

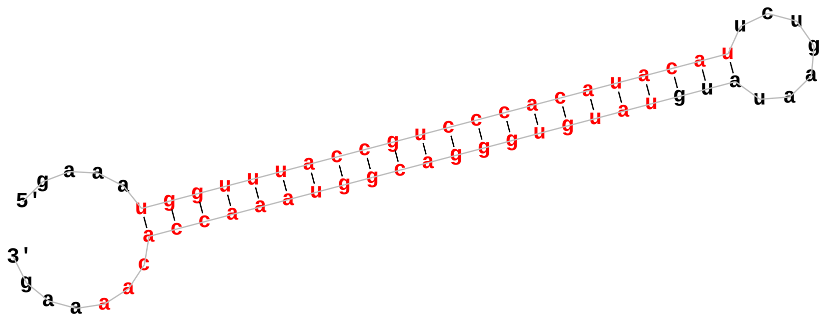

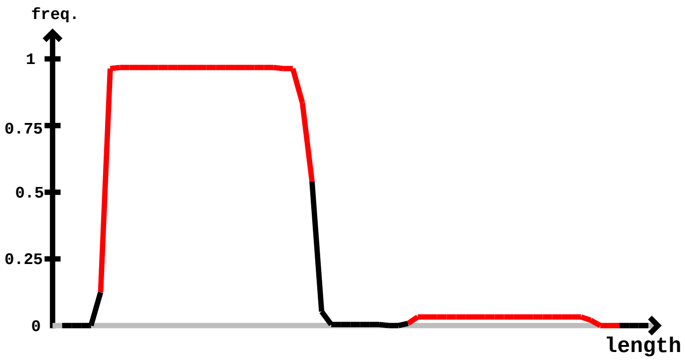


```
        oar-miR-299-5p
                                                oar-miR-299-3p
5'- gaaaugguuuaccgucccacauacauucugaauauguaugugggacgguaaaccacaaaag  -3'   exp
    ....((((((((((((((((((((((.......))))))))))))))))))))))......   reads    mm     sample
    ...augguuuaccgucccacau.......................................   1        0      seq
    ...Gugguuuaccgucccacauac.....................................   1        1      seq
    ...augguuuaccgucccacauac.....................................   5        0      seq
    ...augguuuaccgucccacauaca....................................   24       0      seq
    ....ugguuuaccgucccacauac.....................................   26       0      seq
    ....ugguuuaccgucccacauaca....................................   48       0      seq
    ....ugguuuaccgucccacauacau...................................   121      0      seq
    ....ugguuuaccgucccacauacauu..................................   7        0      seq
    ....ugguuuaccgucccacauacauA..................................   5        1      seq
    ....ugguuuaccgucccacauacauucugaau............................   1        0      seq
    .....gguuuaccgucccacauaca....................................   1        0      seq
    ...................................guaugugggacgguaaacca......   2        0      seq
    ....................................uaugugggacgguaaacc.......   3        0      seq
    ....................................uaugugggacgguaaacca......   3        0      seq
```
